# Supplementary material for: Quantum spin Hall insulators in centrosymmetric thin films composed from topologically trivial BiTeI trilayers
Source: Sci Rep. 2017 Mar 2;7:43666. doi: 10.1038/srep43666 (PMC5333630; doi:10.1038/srep43666)
Supplement: Supplementary Information [file srep43666-s1.pdf]

# Supplementary Information for
# "Quantum spin Hall insulators in centrosymmetric thin films composed from topologically trivial BiTeI trilayers"

I. A. Nechaev,[1,2,3,*] S. V. Eremeev,[2,3,4,5] E. E. Krasovskii,[5,6,7]
P. M. Echenique,[5,6] and E. V. Chulkov[1,2,3,5,6]

[1] *Centro de Física de Materiales CFM - MPC and Centro Mixto CSIC-UPV/EHU, 20018 San Sebastián/Donostia, Spain*

[2] *Tomsk State University, 634050 Tomsk, Russia*

[3] *Saint Petersburg State University, 198504 Saint Petersburg, Russia*

[4] *Institute of Strength Physics and Materials Science, 634055 Tomsk, Russia*

[5] *Donostia International Physics Center, 20018 San Sebastián/Donostia, Spain*

[6] *Departamento de Física de Materiales UPV/EHU, Facultad de Ciencias Químicas, UPV/EHU, Apdo. 1072, 20080 San Sebastián/Donostia, Spain*

[7] *IKERBASQUE, Basque Foundation for Science, 48013 Bilbao, Spain*

[*] swxnenei@ehu.eus

## SUPPLEMENTARY FIGURES

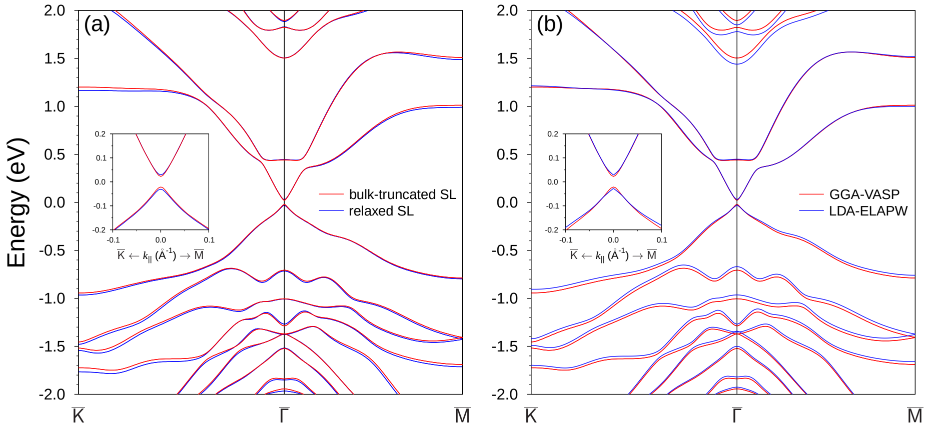


**Supplementary Figure** 1. **Band structure of free-standing SL.** (a) Band structure obtained within the dispersion corrected GGA-VASP (DFT-D3) calculations with and without relaxation. (b) Electronic spectra for bulk-truncated SL calculated within DFT-D3 and LDA-ELAPW. Insets demonstrate magnified view of the gap vicinity.

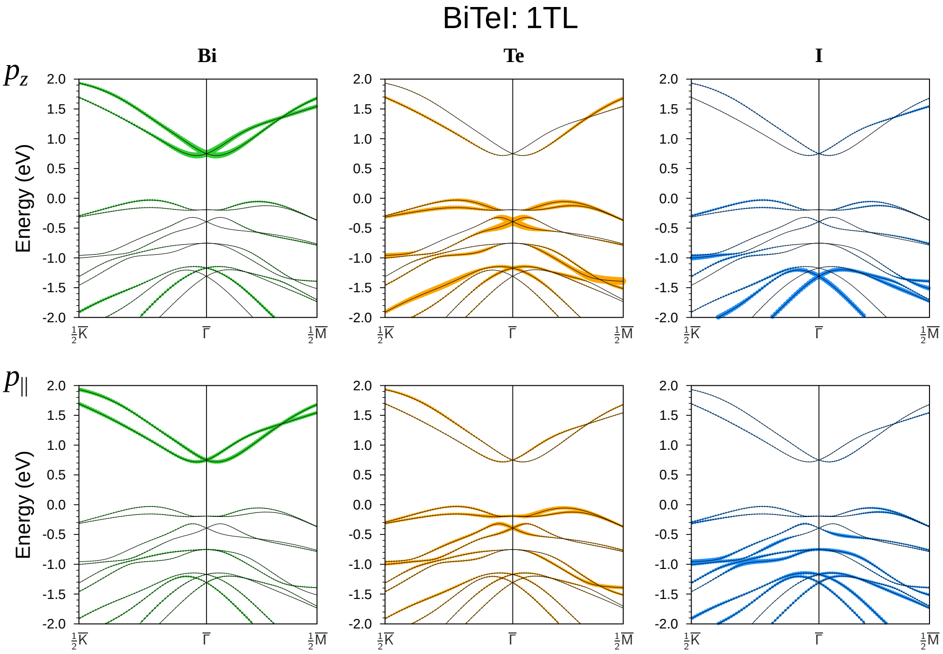


**Supplementary Figure 2. Orbital-resolved band structure of a free-standing BiTeI trilayer (TL).**

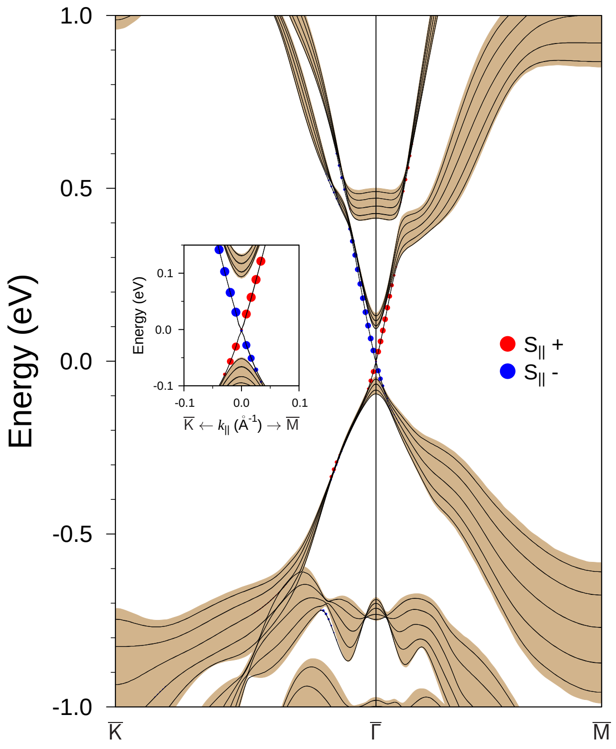


**Supplementary Figure** 3. **Spin-resolved surface electronic structure of $Bi_2Te_2I_2$.** Red and blue circles indicate positive and negative sign of in-plane, $S_{\|}$ spin components, respectively. Shaded area marks projection of the bulk states.

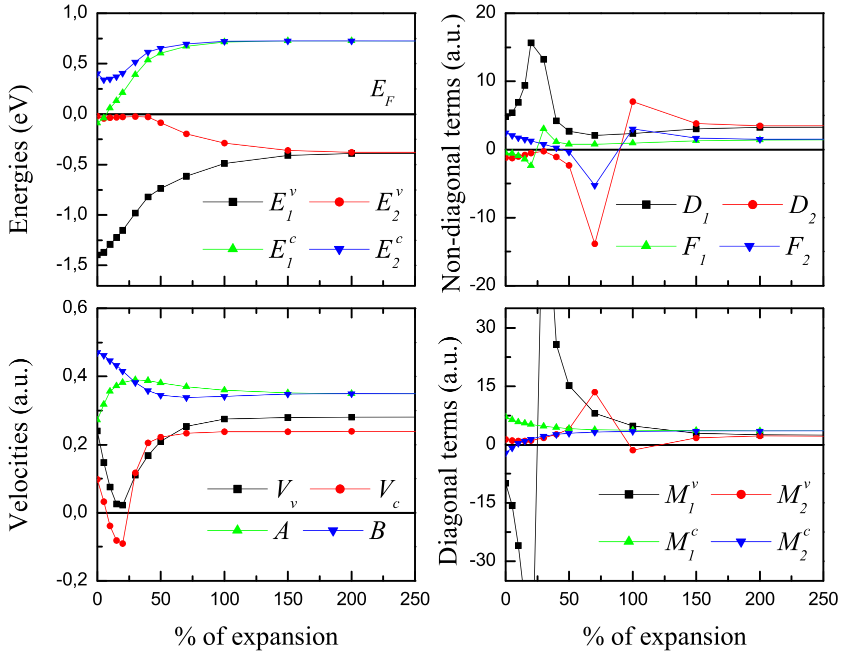


**Supplementary Figure** 4. **The parameters of the eight-band Hamiltonian as a function of the van-der-Waals spacing.** The expansion of the spacing is given in percents of its equilibrium value.

## SUPPLEMENTARY NOTES

### Supplementary Note 1

### Band structure calculations

The LDA band structure is obtained with the extended LAPW (linear augmented plane wave) method[1] using the full potential scheme of Ref. 2. In this method, the extension of the LAPW radial basis set provides a hight accuracy of the band energies and respective wave functions over a wide energy region from the semi-core states to very high energies. As a consequence, in treating the spin-orbit interaction by a second variation method[3] we can include the scalar-relativistic bands up to at least 300 eV to ensure a good convergence of the inverse effective mass, with a deviation from the second derivative of the $E(\mathbf{k})$ curves within 3%.

Additional calculations were carried out within generalized gradient approximation (GGA) with the projector augmented-wave method [6, 7] as realized in the Vienna Ab Initio Simulation Package (VASP) [8, 9]. DFT-D3 van der Walls corrections [10] was applied for accurate structure optimization and further band-structure calculations. Bulk lattice parameters and atomic positions of both BiTeI (the 156 (P3m1) space group) and $Bi_2Te_2I_2$ (the 164 (P$\bar{3}$m1) space group) were optimized. The lattice parameters of BiTeI ($a$ = 4.337 Å, $c$ = 6.850 Å) agree well with experimental values ($a$ = 4.34 Å, $c$ = 6.854 Å). The optimized $a$ and $c$ parameters for $Bi_2Te_2I_2$ bulk have been obtained to equal 4.354 and 13.421 Å, respectively. The interlayer spacings in BiTeI are (in Å): Te-Bi – 1.741, Bi-I – 2.142, I-Te – 2.968. In $Bi_2Te_2I_2$ the Te-Bi and Bi-I spacings have close values to those in BiTeI (1.750 and 2.087, respectively) and Te-Te, and I-I interlayer distances are 2.655 and 3.094 Å, respectively. The total energy of BiTeI, $E_{\mathrm{BiTeI}}$, turned out to be only 0.5 meV (per BiTeI formula unit) lower than that for $Bi_2Te_2I_2$ structure, $\frac{1}{2}E_{\mathrm{Bi_2Te_2I_2}}$. For single free-standing $Bi_2Te_2I_2$ SL we performed additional structural optimization. A small contraction was obtained in the Te-Te distance (2.628 Å) and a small expansion in the outer Bi-I spacing (2.102 Å), while Te-Bi interlayer spacing remained unchanged as compared to the bulk value. Despite these structural changes in the relaxed free-standing SL its electronic spectrum is almost the same as in the bulk-truncated film (Supplementary Figure 1 (a)), especially in the band gap region. For this reason, for electronic structure calculations for thin slabs as

well as for $Bi_2Te_2I_2$ surface, we use the unrelaxed bulk-truncated geometry, while to study the influence of the Te-Te van der Waals spacing expansion in SL on its electronic structure we use the relaxed geometry. The obtained equilibrium structural parameters were taken both for the dispersion corrected GGA and LDA calculations. The electronic spectra for free-standing SL and thicker slabs obtained within these two approximations are almost identical (Supplementary Figure 1 (b)). The analysis of the spatial charge distribution of the Dirac surface state as well as of the spin and spin-orbital textures for $Bi_2Te_2I_2$ surface, shown in Fig. 2 of the main text, has been performed on the base of the dispersion corrected GGA calculations.

## Supplementary Note 2

## Construction of model Hamiltonians

Model Hamiltonians are constructed within the second-order $\mathbf{k}\cdot\mathbf{p}$ perturbation theory, $H_{\mathbf{kp}} = H^{(0)} + H^{(1)} + H^{(2)}$, as an expansion around the point $\mathbf{k} = 0$. The zero-order term is $H^{(0)}_{nimj} = E_n\delta_{mn}\delta_{ij}$, where $E_n$ is the doubly degenerate band energy with two orthogonal wave functions $\Psi_{n1}$ and $\Psi_{n2}$ (Kramers pair, hereafter $\Psi_{ni}$) due to inversion symmetry of the systems under study. The linear term is defined as

$$H^{(1)}_{nimj} = \frac{\hbar}{m_0}\mathbf{k}\cdot\boldsymbol{\pi}_{nimj}$$

with the matrix elements[4] $\boldsymbol{\pi}_{nimj} = \langle\Psi_{ni}|\boldsymbol{\pi}|\Psi_{mj}\rangle$ of the relativistic velocity operator $\boldsymbol{\pi} = -i\hbar\boldsymbol{\nabla} + \hbar\left[\boldsymbol{\sigma}\times\boldsymbol{\nabla}V\right]/4m_0c^2$, where $\boldsymbol{\sigma} = (\sigma_x, \sigma_y, \sigma_z)$ is the vector of Pauli matrices, $V(\mathbf{r})$ is the crystal potential. The second-order term is given by

$$H^{(2)}_{nimj} = \frac{\hbar^2k^2}{2m_0}\delta_{mn}\delta_{ij} + \frac{\hbar^2}{m_0^2}\sum_{\alpha\beta}k_\alpha D^{\alpha\beta}_{nimj}k_\beta,$$

where $\alpha, \beta = x, y, z$ and (see, e.g., Ref. 5)

$$D^{\alpha\beta}_{nimj} = \frac{1}{2}\sum_{n'i'}\pi^{\alpha}_{nin'i'}\pi^{\beta}_{n'i'mj}\left(\frac{1}{E_n - E_{n'}} + \frac{1}{E_m - E_{n'}}\right).$$

Here, the index $n'$ runs over all the calculated relativistic bands (from semi-core levels up to high-lying unoccupied bands) excluding the chosen $\mathbf{k}\cdot\mathbf{p}$ basis set (the so-called Löwdin's partitioning[5]).

The *ab initio* wave functions $\Psi_{ni}$ numerically obtained with the extended LAPW method are arbitrarily ordered and have phases of no physical meaning. These phases, nevertheless, affect $H_{\mathbf{kp}}$ non-diagonal elements. Therefore, to keep the form of the $\mathbf{k}\cdot\mathbf{p}$ Hamiltonian in different calculations with the continuously varying geometry (increasing the van-der-Waals spacing) and for different number of the sextuple layers (changes in the film thickness), we first transfer to the basis that diagonalizes the $z$-component of the total angular momentum $\mathbf{J} = \mathbf{L}+\mathbf{S}$ in the atomic sphere that has the largest weight in the $n$-th band, $\Psi_{n1(2)} \to \Psi_{n\uparrow(\downarrow)}$. Next, we adjust the phases of the new basis functions to explicitly form the Kramers pair $\Psi_{n\downarrow} = \hat{T}\Psi_{n\uparrow}$ with the time reversal operator $\hat{T} = Ki\sigma_y$, where $K$ is the complex conjugate operator. Finally, we turn the wave-function phases of the $n$-th and $m$-th pairs of different parity to get $i\pi^{x(z)}_{n\uparrow m\downarrow}$ real.

**Supplementary Note 3**

**Matrix form of the four- and eight-band k·p Hamiltonians**

For the bulk $Bi_2Te_2I_2$, in the basis $\Psi^{\text{bulk}}_{v\uparrow}$, $\Psi^{\text{bulk}}_{v\downarrow}$, $\Psi^{\text{bulk}}_{c\uparrow}$, $\Psi^{\text{bulk}}_{c\downarrow}$, where $v$ and $c$ stand for the valence and conduction band, respectively, the four-band Hamiltonian is given by

$$H^{\text{bulk}}_{\mathbf{kp}} = CI + \begin{pmatrix} M & 0 & iV_z k_z & -iV_\parallel k_- \\ 0 & M & iV_\parallel k_+ & iV_z k_z \\ -iV_z k_z & -iV_\parallel k_- & -M & 0 \\ iV_\parallel k_+ & -iV_z k_z & 0 & -M \end{pmatrix},$$

where $I$ is the 4×4 identity matrix, $C = C_0 + C_z k_z^2 + C_\parallel k_\parallel^2$, $M = M_0 + M_z k_z^2 + M_\parallel k_\parallel^2$, $k_\parallel^2 = k_x^2 + k_y^2$, and $k_\pm = k_x \pm ik_y$.

In finite slab geometry calculations, for all the films considered the Hamiltonian is derived in the basis $\Psi^{\text{slab}}_{v\uparrow}$, $\Psi^{\text{slab}}_{c\downarrow}$, $\Psi^{\text{slab}}_{c\uparrow}$, $\Psi^{\text{slab}}_{v\downarrow}$ and is given by the matrix

$$H^{\text{slab}}_{\mathbf{kp}} = CI + \begin{pmatrix} M & -iV_\parallel k_- & 0 & 0 \\ iV_\parallel k_+ & -M & 0 & 0 \\ 0 & 0 & -M & -iV_\parallel k_- \\ 0 & 0 & iV_\parallel k_+ & M \end{pmatrix},$$

where $C = C_0 + C_\parallel k_\parallel^2$ and $M = M_0 + M_\parallel k_\parallel^2$.

The eight-band Hamiltonian as obtained in the basis $\Psi^{\text{slab}}_{v_1\uparrow}$, $\Psi^{\text{slab}}_{v_2\downarrow}$, $\Psi^{\text{slab}}_{c_1\uparrow}$, $\Psi^{\text{slab}}_{c_2\downarrow}$, $\Psi^{\text{slab}}_{c_2\uparrow}$, $\Psi^{\text{slab}}_{c_1\downarrow}$, $\Psi^{\text{slab}}_{v_2\uparrow}$, $\Psi^{\text{slab}}_{v_1\downarrow}$ (the parity is (+), (−), (+), and (−) for $v_1$, $v_2$, $c_1$, and $c_2$ Kramers pairs, respectively) reads as

$$H^{1\text{SL}}_{\mathbf{kp}} = \begin{pmatrix} E_1^v + M_1^v k_\parallel^2 & -iV_v k_- & D_1 k_\parallel^2 & iAk_- & 0 & F_1 k_+^2 & 0 & 0 \\ iV_v k_+ & E_2^v + M_2^v k_\parallel^2 & -iBk_+ & D_2 k_\parallel^2 & -F_2 k_-^2 & 0 & 0 & 0 \\ D_1 k_\parallel^2 & iBk_- & E_1^c + M_1^c k_\parallel^2 & iV_c k_- & 0 & 0 & 0 & -F_1 k_+^2 \\ -iAk_+ & D_2 k_\parallel^2 & -iV_c k_+ & E_2^c + M_2^c k_\parallel^2 & 0 & 0 & F_2 k_-^2 & 0 \\ 0 & -F_2 k_+^2 & 0 & 0 & E_2^c + M_2^c k_\parallel^2 & iV_c k_- & D_2 k_\parallel^2 & iAk_- \\ F_1 k_-^2 & 0 & 0 & 0 & -iV_c k_+ & E_1^c + M_1^c k_\parallel^2 & -iBk_+ & D_1 k_\parallel^2 \\ 0 & 0 & 0 & F_2 k_+^2 & D_2 k_\parallel^2 & iBk_- & E_2^v + M_2^v k_\parallel^2 & -iV_v k_- \\ 0 & 0 & -F_1 k_-^2 & 0 & -iAk_+ & D_1 k_\parallel^2 & iV_v k_+ & E_1^v + M_1^v k_\parallel^2 \end{pmatrix}.$$

To clearly demonstrate the quantum topological phase transition, in our eight-band $\mathbf{k}{\cdot}\mathbf{p}$ description of the 1SL film in the topologically non-trivial state we chose the $v_2$ and $c_1$ basis wave functions to be correspond to the lowest conduction band and the highest valence band, respectively. For this state, we have $M_2^v \approx C_\parallel - M_\parallel$, $M_1^c \approx C_\parallel + M_\parallel$, and the coupling of the $v_2$ and $c_1$ bands $B = V_\parallel$, where $C_\parallel$, $M_\parallel$, and $V_\parallel$ are the parameters of the 4-band Hamiltonian.

The parameters of the eight-band Hamiltonian are shown in Supplementary Figure 4 as a function of the van-der-Waals spacing. Note that in contrast to a tight-binding consideration, there are no interband coupling parameters vanishing at a very large distance between two BiTeI trilayers forming the studied sextuple layer. It is worth mentioning the behaviour of the parameters $V_v$, $V_c$, $A$, and $B$. The first two parameters, $V_v$ and $V_c$, coupling the bands within the pairs $v_1 \leftrightarrow v_2$ and $c_1 \leftrightarrow c_2$, respectively, have a deep minimum at $\sim 25\%$ of expansion. Such a rather big loss of coupling is accompanied by a slightly increased coupling between the pairs ($A$ and $B$).

Finally, we consider the upper-left $4\times4$ block of the above 8-band Hamiltonian, analysing its parameters in the limit of the well-separated BiTeI TLs. For simplicity, we fix the velocities $V_v = V_c \equiv \alpha$ and the inverse effective masses $M_1^v = M_2^v = M_1^c = M_2^c \equiv m_\parallel$. Additionally, we take into account that in the limit considered $D_1 = D_2 \equiv D$ and $A = B \equiv v_\parallel$. As a result, we have the model Hamiltonian of the following compact form

$$\begin{aligned} h_{\mathrm{TL}} = M_0\tau_z\sigma_0 + m_\parallel\tau_0\sigma_0 k_\parallel^2 - \alpha\tau_z(\sigma_x k_y - \sigma_y k_x) \\ + D\tau_x\sigma_0 k_\parallel^2 + v_\parallel\tau_x(\sigma_x k_y - \sigma_y k_x), \end{aligned} \tag{1}$$

which is diagonalized with energy bands given by

$$E_{s\mu}(k_\parallel) = m_\parallel k_\parallel^2 + s\sqrt{(\alpha k_\parallel + \mu|M_0|)^2 + (v_\parallel k_\parallel + \mu D k_\parallel^2)^2}$$

with $s, \mu = \pm1$. The Hamiltonian $h_{\mathrm{TL}}$ can be attributed to a free-standing TL, formed by two "Rashba 2D electron systems" (the first line in the above equation) referred to by $\boldsymbol{\tau}$. These systems lying at different energies, $\pm M_0$, are characterized by the same inverse effective mass, $m_\parallel$, and by the equal strengths, $\alpha$, of the Rashba spin-orbit interaction but of different sign. The systems are coupled through the linear and quadratic (in $\mathbf{k}$) terms (the second line). The above $h_{\mathrm{TL}}$ can be used to construct a Hamiltonian describing the non-trivial SL within a tight-binding-like technique. Actually, with the spin-conserving hopping

parameter $t_z$ of $\sim 0.15$ eV for the Bi-atomic layers ($-M_0$) and at least three times larger for the adjacent Te-atomic layers ($+M_0$) we can reproduce the spectrum of the SL with the inverted band gap in the vicinity of the point $\mathbf{k} = 0$. Iterating this SL block with proper parameters of tunneling between them, we arrive at the gapless Dirac surface states. It is worth noting that the $\mathbf{k}$-linear term with the parameter $v_{\|}$ plays crucial role in formation of this states.

## SUPPLEMENTARY REFERENCES


[1] E. E. Krasovskii, *Accuracy and convergence properties of the extended linear augmented-plane-wave method*, Phys. Rev. B **56**, 12866 (1997).

[2] E. E. Krasovskii, F. Starrost, and W. Schattke, *Augmented Fourier components method for constructing the crystal potential in self-consistent band-structure calculations*, Phys. Rev. B **59**, 10504 (1999).

[3] D. D. Koelling and B. N. Harmon, *A technique for relativistic spin-polarised calculations*, J.Phys. C: Solid State Phys. **10**, 3107 (1977).

[4] E. E. Krasovskii, *Microscopic origin of the relativistic splitting of surface states*, Phys. Rev. B **90**, 115434 (2014).

[5] L. C. Lew Yan Voon and M. Willatzen, *The k·p Method: Electronic Properties of Semiconductors* (Springer-Verlag, Berlin, 2009).

[6] P.E. Blöchl, *Projector augmented-wave method*, Phys. Rev. B **50**, 17953 (1994).

[7] G. Kresse, D. Joubert, *From ultrasoft pseudopotentials to the projector augmented-wave method*, Phys. Rev. B **59**, 1758 (1999).

[8] G. Kresse, J. Hafner, *Ab initio molecular dynamics for open-shell transition metals*, Phys. Rev. B **48**, 13115 (1993).

[9] G. Kresse, J. Furthmüller, *Efficient iterative schemes for ab initio total-energy calculations using a plane-wave basis set*, Phys. Rev. B **54**, 11169 (1996).

[10] S. Grimme, J. Antony, S. Ehrlich, and H. Krieg, *A consistent and accurate ab initio parametrization of density functional dispersion correction (DFT-D) for the 94 elements H-Pu*, J. Chem. Phys. **132**, 154104 (2010).
